# MicroRNA-429 inhibits cancer cell proliferation and migration by targeting AKT1 in renal cell carcinoma

**DOI:** 10.3892/mco.2020.2028

**Published:** 2020-04-06

**Authors:** Zhengming Su, Ganggang Jiang, Jinlan Chen, Xing Liu, Haibo Zhao, Zhiyuan Fang, Yongzhong He, Xianhan Jiang, Guibin Xu

Mol Clin Oncol 12:75-80, 2020; DOI: 10.3892/mco.2019.1940

Following the publication of this article, the authors have realized that one of the grant numbers published in the ‘Funding’ section of their paper had been written incorrectly: The grant no. for the granted received from the Medical Science and Technology Program of Guangzhou of China should have been written as “20171A011**329**” instead of “20171A011092”. Therefore, the ‘Funding’ section of the Declarations for this paper should have been written as follows:

**Funding**

The current work was supported by funding from Science and Technology Plan Project of Guangzhou of China (grant no. 201510010177), Medical Science and Technology Program of Guangzhou of China (grant no. 20171A011**329** and 20171A010325), Youth Scientific Research Project of Guangzhou Medical University of China (grant no. 2015A14) and the Medical Research Foundation of Guangdong Province of China (grant no. A2018093).

The authors apologize to the funders of their research project, and to the readership of the Journal for any inconvenience caused.

